# Supplementary material for: Number and dissimilarity of global change factors influences soil properties and functions
Source: Nat Commun. 2024 Sep 18;15:8188. doi: 10.1038/s41467-024-52511-2 (PMC11410830; doi:10.1038/s41467-024-52511-2)
Supplement: Supplementary file 1 — Supplementary Information [file 41467_2024_52511_MOESM1_ESM.pdf]

Supplementary Information for

Factor number and dissimilarity drive effects of multiple global change factors on soil properties and functions

This file includes:

Supplementary Figures S1 to S8

Supplementary References

Supplementary Fig. 1. Calculating factor distances and classifying factors by seven soil responses. a Heatmap of mean responses of soil properties to each single factor applied alone. In the dendrogram, single factors are clustered by the Euclidean similarity of scaled soil responses. **b** Principal coordinates analysis (PCoA) shows the Euclidean distances between single factors.

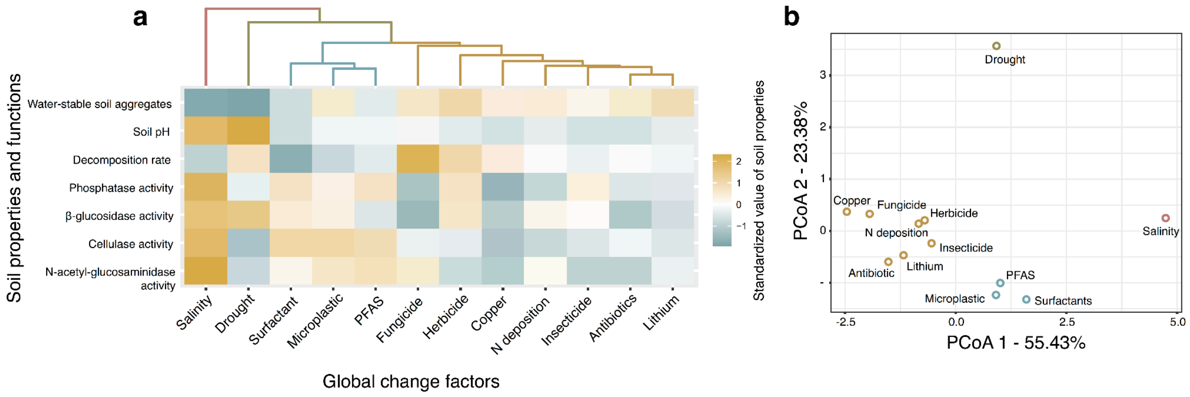

Supplementary Fig. 2. Structure of hierarchical modeling framework for hypothesis testing. a Structure of hierarchical modeling framework. Different compositions of predictors are included in Model 1 to 7. Model complexity increases when more predictors are included. b Testing hypothesis by comparing models. Predictors for testing hypotheses include number of factors and factor dissimilarity index. Factor composition of every treatment (coded by 0/1) theoretically includes all the information from the experimental design. In the machine learning algorithm, including factor composition information enables the model to have the best fit to the experimental data, but it can not provide resolution in terms of mechanism.

a

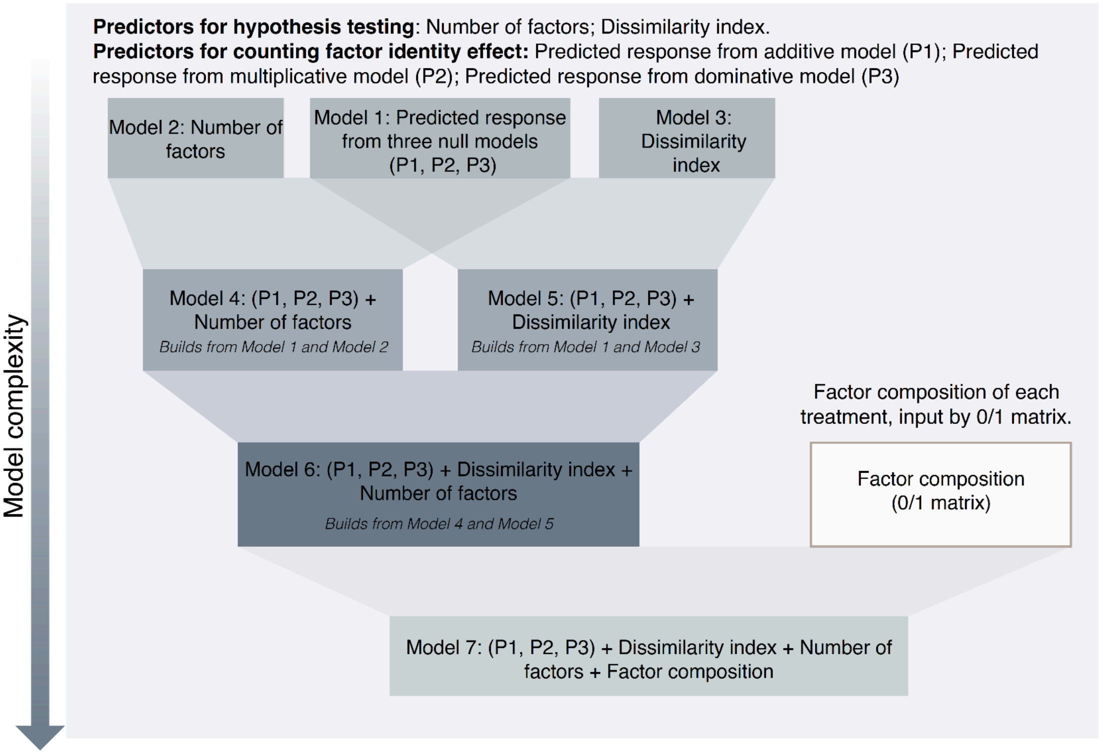

b **Testing hypothesis by comparing models:**

Model 1: Variability of soil response to multiple GCFs explained by three null model predictions, which is regarded as the factor identity effect.

Model 2: Variability of soil responses to multiple GCFs explained by number of factor effect.

Model 3: Variability of soil responses to multiple GCFs explained by factor dissimilarity indices.

Model 4 → Model 1: Variability of soil responses to multiple GCFs further explained by number of factor effect on the basis of including factor identity effect.

Model 5 → Model 1: Variability of soil responses to multiple GCFs further explained by factor dissimilarity effect on the basis of including factor identity effect.

Model 6 → Model 4: Variability of soil responses to multiple GCFs further explained by factor dissimilarity effect on the basis of including factor identity effect and number of factor effect.

Model 6 → Model 5: Variability of soil responses to multiple GCFs further explained by number of factor effect on the basis of including factor identity effect and factor dissimilarity effect.

Model 7 → Model 6: Variability of soil responses to multiple GCFs further explained by factor composition information on the basis of including all hypothetical predictors and factor identity effect.

Supplementary Fig. 3. Explained variance of soil properties and functions by seven models from the hierarchical modeling framework. Explained variance of soil N-acetyl-glucosaminidase activity (a), cellulase activity (b), β-glucosidase activity (c), phosphatase activity (d), decomposition rate (e), soil pH (f) and WSA (g) by seven models from the hierarchical modeling framework.

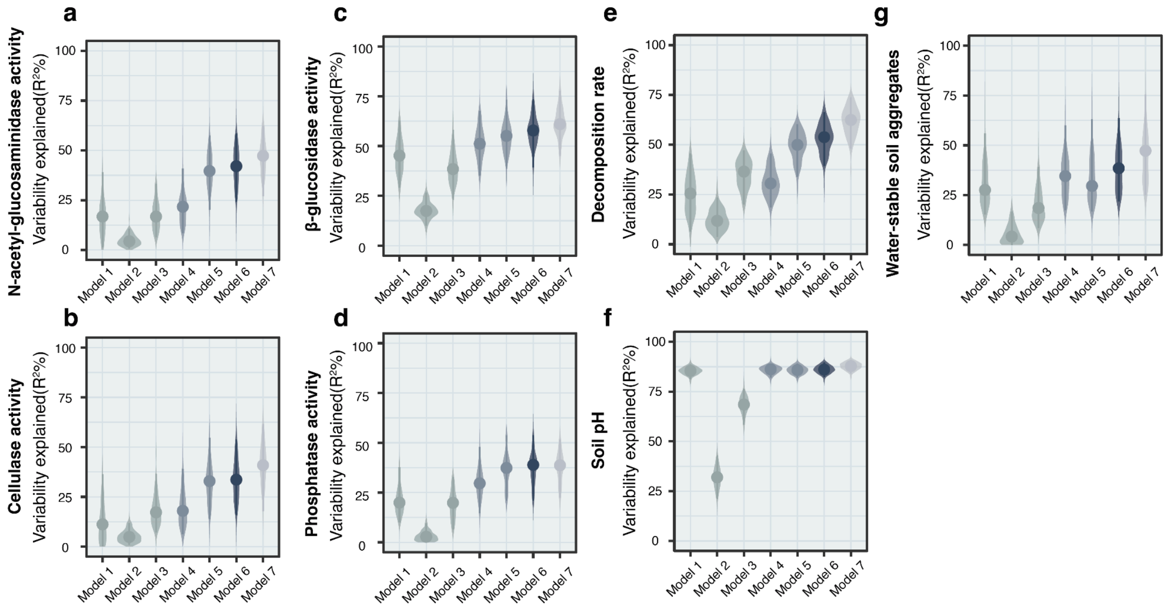

Supplementary Fig. 4. Rescaled deviation of soil responses (soil decomposition rate, soil pH and water-stable soil aggregates) from three null model predictions with different numbers of factors (factor = 2, 5 or 8). [a (1) to c (3)] Scatter points represent the standardized deviation of soil responses from null model predictions for multiple-factor treatments. Net interaction type of each multiple-factor treatment is marked as different colored points (antagonistic, blue; synergistic, red; no interaction, gray). The best-fitting null model for each soil response has been selected based on the smallest model sum of squared deviation (SSD), and it is indicated by the bold frame [a (1), b (3) and c (3)]. The overall deviances of soil responses from null models in three number of factor groups (factor = 2, 5 and 8) are shown by boxplot. The significant deviations from zero were evaluated by two sided t-tests (* 0.01 < P ≤ 0.05; ** 0.001 < P ≤ 0.01; *** P ≤ 0.001).

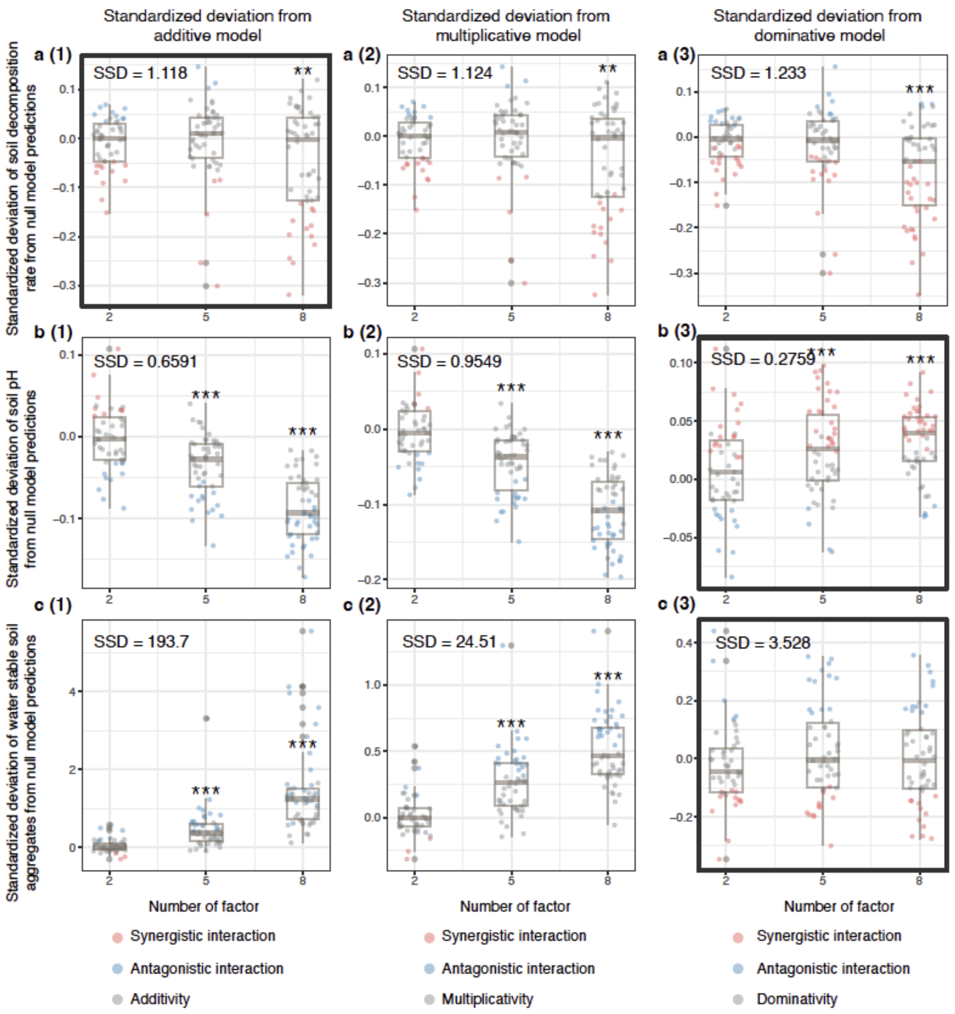

Supplementary Fig. 5. Rescaled deviation of soil enzymatic activity from three null model predictions for different numbers of factors (factor = 2, 5 or 8). [a (1) to c (3)] Scatter points represent the standardized deviation of soil enzymatic activity from null model predictions for multiple-factor treatments. Net interaction type of each multiple-factor treatment is marked as different colored points (antagonistic, blue; synergistic, red; no interaction, gray). The best-fitting null model for each soil enzymatic activity has been selected based on the smallest model sum of squared deviation (SSD), and it is indicated by the bold frame [a (2), b (3), c (2) and d (2)]. The overall deviances of soil enzymatic activity from null models in three number of factor groups (factor = 2, 5 and 8) are shown by boxplot. The significant deviations from zero were evaluated by two sided t-tests (*0.01 < P ≤ 0.05; ** 0.001 < P ≤ 0.01; *** P ≤ 0.001).

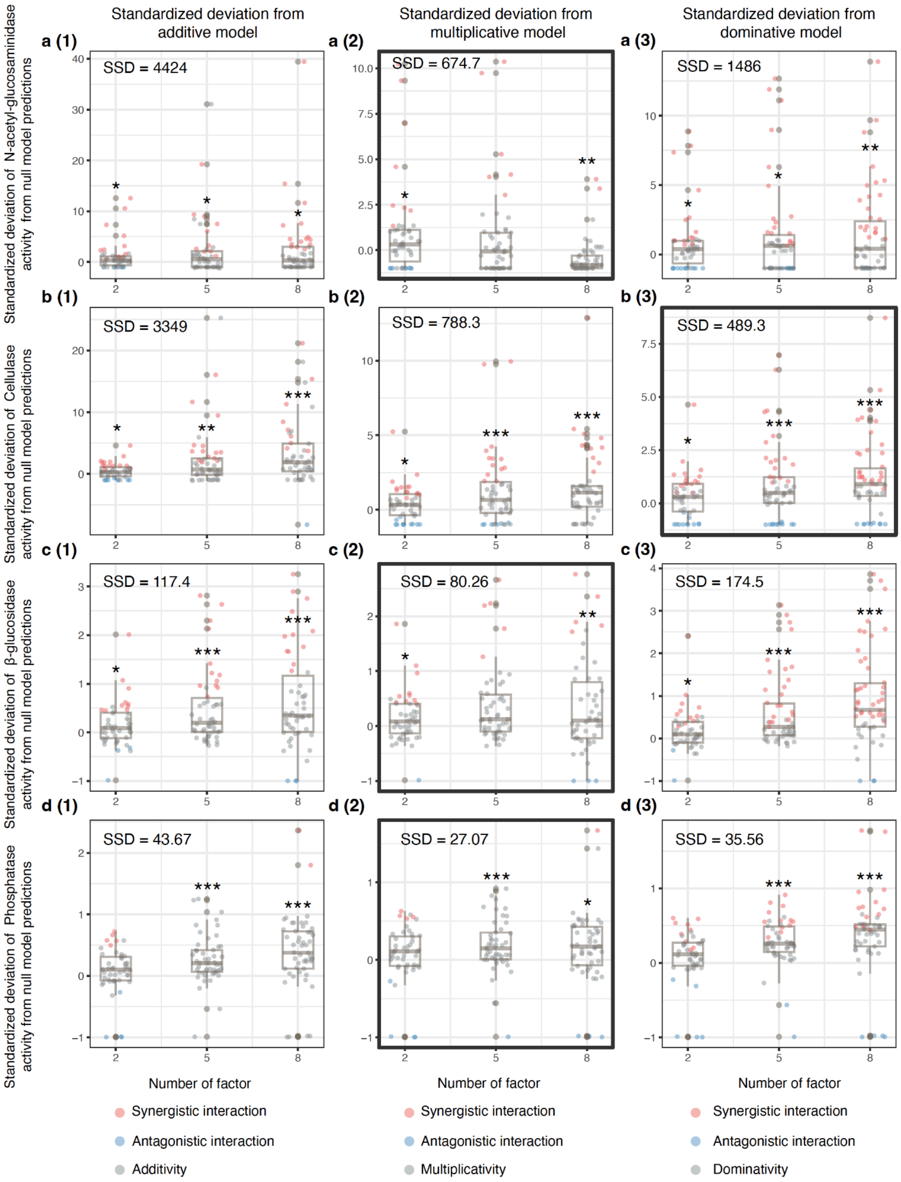

Supplementary Fig. 6. Effects of organic solvents on soil properties and functions, including N-acetyl-glucosaminidase activity (a), cellulase activity (b), glucosidase activity (c), phosphatase activity (d), decomposition rate (e), soil pH (f) and water stable soil aggregates (g). Illustrated are effect sizes of organic solvents (DMSO and acetone), that are used in experimental treatments for dissolving chemical factors (herbicide and fungicide), on soil properties and functions. Tested groups are Control Treatment (CT, n=20, applied by corresponding solvents including organic solvents) and Water Control Treatment (WC, n=10, organic solvents were replaced by the same amount of water). CT and WC treatments are incubated together with other experimental treatments in the same condition, and the soil properties and functions of those treatments are measured also at the same time with other treatments.

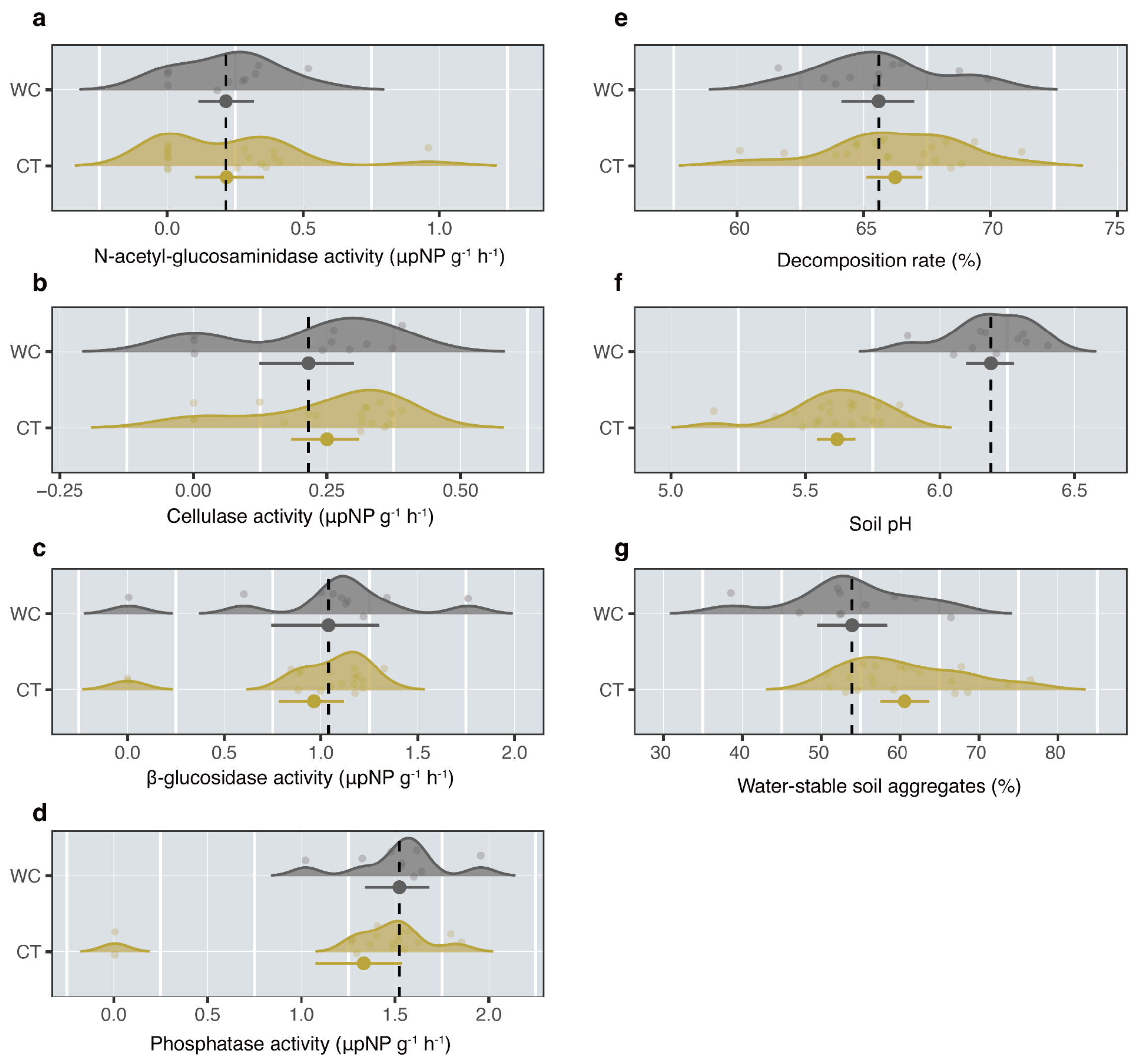

Supplementary Fig. 7. Density distribution of factor dissimilarity indices of multiple-factor treatments in different number of factor groups normalized separately for each number of factor level (a) and normalized globally (b). P values obtained from pairwise t-tests are shown between every number of factor groups. In panel (a) and (b) we compared the difference of normalizing the dissimilarity index in each factor level and normalizing it globally. When dissimilarity indices are normalized in each number of factor level, their distributions are significantly different from each other (P < 0.05), which means the dissimilarity indices co-vary with factor levels. We think this bias partially comes from the random selection method (for example we may select more 2-factor combinations with lower dissimilarity by chance) and partially comes from the intrinsic unevenness of distances among 12 single factors. Even though we can not fully disentangle the dissimilarity indices from co-varying with factor level, our further approaches enable us to disentangle the effects of factor dissimilarity through hierarchical modeling methods. These methods enable us to assess the unique contribution of factor dissimilarity to the model predictability by comparing to a model taking into account the number of factor effects. However, when dissimilarity indices are normalized globally, even though their mean values are not different from each other (P > 0,05), their distributions are quite different for different factor groups, for example, the range for 2-factor group is 1 but the range for 8-factor group is 0.259. These widely varying ranges will interfere with our further analysis for assessing the effect of factor dissimilarity. Due to the aforementioned reasons, we opted to utilize dissimilarity indices normalized within each factor level for our analysis.

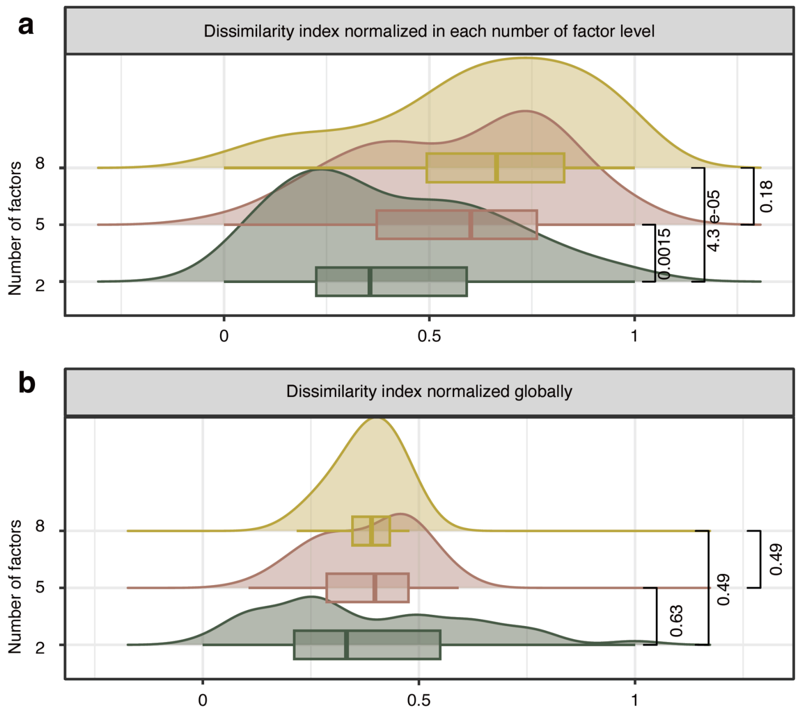

Supplementary Fig. 8. A comparison of dendrograms of 12 GCFs clustered based on their effects on soil properties and functions (measured in this experiment) (a) and based on a priori trait-based classification from expert opinions (b). Cophenetic correlation between two trees was calculated based on a bootstrapping method developed by a previous study[24]. The Cophenetic correlation test calculates the correlation between two cophenetic distance matrices of the two trees. The value of the cophenetic correlation coefficient can range between -1 to 1. With near 0 values meaning that the two trees are not statistically similar. After applying 1,000 times of permutation, the results show that the two dendrograms have structures more similar to each other than expected by chance (cophenetic correlation coefficient: mean = 0.2869 [95%CI: 0.073-0.475] > 0), indicating a priori trait-based ordering of factors having similar traits affecting soil properties and functions. It may provide advantages for future GCF management evaluating factor dissimilarity based on the priori trait-based factor classification systems.

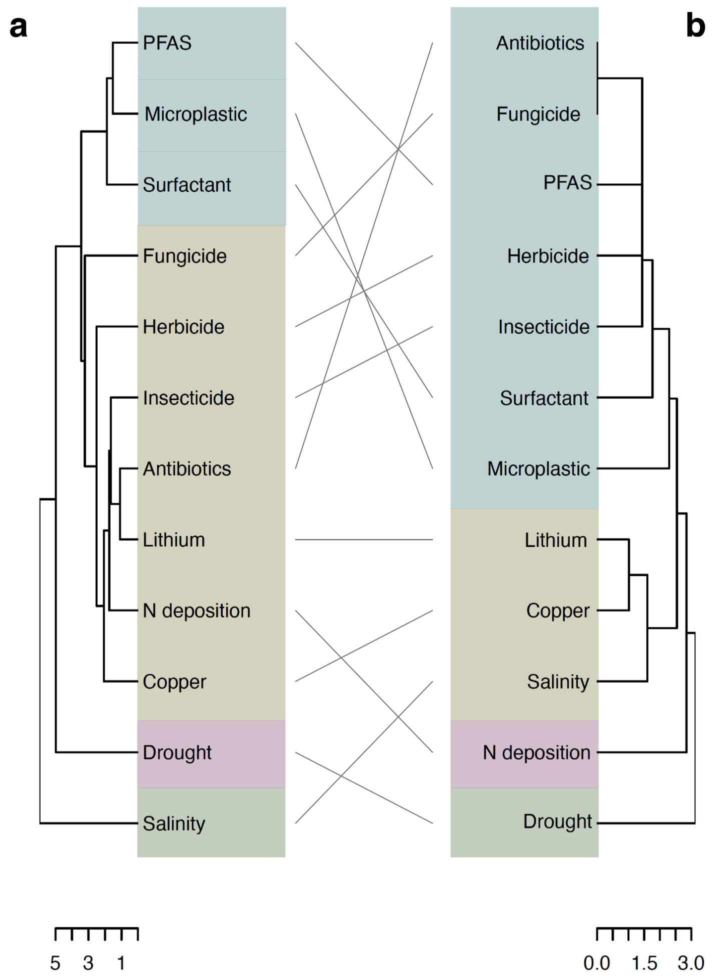

References

1. Rillig, M. C., Ryo, M. & Lehmann, A. Classifying human influences on terrestrial ecosystems. *Glob. Change Biol.* **27**, 2273–2278 (2021).
