# Supplementary material for: Number and dissimilarity of global change factors influences soil properties and functions
Source: Nat Commun. 2024 Sep 18;15:8188. doi: 10.1038/s41467-024-52511-2 (PMC11410830; doi:10.1038/s41467-024-52511-2)
Supplement: Supplementary file 3 — Description of Additional Supplementary Files [file 41467_2024_52511_MOESM3_ESM.pdf]

Supplementary Data 1. Significance test for the effects of single GCFs on soil responses.
Adjusted P values based on Benjamini-Hochberg method obtained from two sided t-tests between each treatment group and controls. (Significant differences with 0.01 < P 0.05 are shown in bold, and P 0.01 are marked by * additionally.)

Supplementary Data 2. Significance test for the effects of groups in different number of factors on soil responses.
Adjusted P values based on the Benjamini-Hochberg method obtained from two sided t-tests between each group and controls. (n indicates the sample size of each group. Significant differences with 0.01 < P 0.05 are shown in bold, and P 0.01 are marked by * additionally.)

Supplementary Data 3. Mean value and 95% confidence interval of R-squared (%) explained by seven random forest models of soil decomposition rate, soil pH and water-stable soil aggregation.

Supplementary Data 4. Mean value and 95% confidence interval of R-squared (%) explained by seven random forest models of soil four enzymatic activities.

Supplementary Data 5. Significance tests for importance measures of predictors of random forest models.
Adjusted P values for relative importance of model predictors calculated from a permutation-based random forest model approach based on the Bonferroni method obtained from two sided t-tests between predictors. (P1 indicates the predicted response from the additive model; P2 indicates the predicted response from the multiplicative model; P3 indicates the predicted response from the dominative model; Significant differences with 0.01 < P 0.05 are shown in bold, and P 0.01 are marked by * additionally.)

Supplementary Data 6. Model formulas for Hierarchical modeling analysis based on general linear models and random forest models.
Model variable P1 indicates the predicted response from the additive model; P2 indicates the predicted response from the multiplicative model; P3 indicates the predicted response from the dominative model.

Supplementary Data 7. Contributions of model predictors on soil decomposition rate, soil pH and water-stable soil aggregates based on general linear models.
The formulas of seven linear models are shown in Supplementary Table 4, and each model is evaluated by its AIC (Akaike information criterion) and log-likelihood (logLik). To test the hypothesis by general linear models, we made five model comparisons based on ANOVA test. The statistical results of model comparisons are provided as the P values and changes of model adjusted R-squared. The + in brackets after the model adjusted R-squared indicate the better model performance than its reference model. Significant differences with 0.01 < P 0.05 are shown in bold, and P 0.01 are marked by * additionally. The statistical test used was two-sided.

Supplementary Data 8. Contributions of model predictors on soil enzymatic activities based on general linear models.

The formulas of seven linear models are shown in Supplementary Table 4, and each model is evaluated by its AIC (Akaike information criterion) and log-likelihood (logLik). To test the hypothesis by general linear models, we made five model comparisons based on ANOVA test. The statistical results of model comparisons are provided as the P values and changes of model adjusted R-squared. The + in brackets after the model adjusted R-squared indicate the better model performance than its reference model. Significant differences with 0.01 < P 0.05 are shown in bold, and P 0.01 are marked by * additionally. The statistical test used was two-sided.

Supplementary Data 9. Statistical assessment of rescaled multi-factor treatment response deviations from three null model predictions.

We provide the deviation mean and sum of squared deviation of the 150 multi-factor treatments from additive, multiplicative and dominative models. The smallest model sum of squared deviation indicates it is the best-fitting null model among all three null models for predicting the responses of corresponding soil measurement, which is shown in bold.

Supplementary Data 10. Significance tests for rescaled deviations for multi-factor treatment responses from three null model predictions for each number of factor level.

P values obtained from two sided t-tests were calculated for the rescaled deviations of each number of factor group compared to zero. (n indicates the sample size of each group. Significant differences with 0.01 < P 0.05 are shown in bold, and P 0.01 are marked by * additionally.)
