# Supplementary material for: Number and dissimilarity of global change factors influences soil properties and functions
Source: Nat Commun. 2024 Sep 18;15:8188. doi: 10.1038/s41467-024-52511-2 (PMC11410830; doi:10.1038/s41467-024-52511-2)
Supplement: Supplementary file 5 — Reporting Summary [file 41467_2024_52511_MOESM5_ESM.pdf]

nature portfolio

Corresponding author(s): Matthias C. Rillig

Last updated by author(s): Jul 25, 2024

Reporting Summary

Nature Portfolio wishes to improve the reproducibility of the work that we publish. This form provides structure for consistency and transparency in reporting. For further information on Nature Portfolio policies, see our Editorial Policies and the Editorial Policy Checklist.

Statistics

For all statistical analyses, confirm that the following items are present in the figure legend, table legend, main text, or Methods section.

n/a	Confirmed	
☐	☒	The exact sample size (n) for each experimental group/condition, given as a discrete number and unit of measurement
☐	☒	A statement on whether measurements were taken from distinct samples or whether the same sample was measured repeatedly
☐	☒	The statistical test(s) used AND whether they are one- or two-sided<br>*Only common tests should be described solely by name; describe more complex techniques in the Methods section.*
☒	☐	A description of all covariates tested
☐	☒	A description of any assumptions or corrections, such as tests of normality and adjustment for multiple comparisons
☐	☒	A full description of the statistical parameters including central tendency (e.g. means) or other basic estimates (e.g. regression coefficient) AND variation (e.g. standard deviation) or associated estimates of uncertainty (e.g. confidence intervals)
☐	☒	For null hypothesis testing, the test statistic (e.g. F, t, r) with confidence intervals, effect sizes, degrees of freedom and P value noted<br>*Give P values as exact values whenever suitable.*
☒	☐	For Bayesian analysis, information on the choice of priors and Markov chain Monte Carlo settings
☒	☐	For hierarchical and complex designs, identification of the appropriate level for tests and full reporting of outcomes
☐	☒	Estimates of effect sizes (e.g. Cohen's d, Pearson's r), indicating how they were calculated

Our web collection on statistics for biologists contains articles on many of the points above.

Software and code

Policy information about availability of computer code

Data collection	No software was used for data collection
Data analysis	All data analyses were performed using R version 4.1.1. The R script is available in a publicly accessible database (https://figshare.com/account/articles/25111388).

For manuscripts utilizing custom algorithms or software that are central to the research but not yet described in published literature, software must be made available to editors and reviewers. We strongly encourage code deposition in a community repository (e.g. GitHub). See the Nature Portfolio guidelines for submitting code & software for further information.

Data

Policy information about availability of data

All manuscripts must include a data availability statement. This statement should provide the following information, where applicable:

- Accession codes, unique identifiers, or web links for publicly available datasets
- A description of any restrictions on data availability
- For clinical datasets or third party data, please ensure that the statement adheres to our policy

All datasets that support the findings of this study have been deposited in the figshare: https://figshare.com/account/articles/25111448.

Research involving human participants, their data, or biological material

Policy information about studies with human participants or human data. See also policy information about sex, gender (identity/presentation), and sexual orientation and race, ethnicity and racism.

Reporting on sex and gender	NA
Reporting on race, ethnicity, or other socially relevant groupings	NA
Population characteristics	NA
Recruitment	NA
Ethics oversight	NA

Note that full information on the approval of the study protocol must also be provided in the manuscript.

Field-specific reporting

Please select the one below that is the best fit for your research. If you are not sure, read the appropriate sections before making your selection.

☐ Life sciences ☐ Behavioural & social sciences ☒ Ecological, evolutionary & environmental sciences

For a reference copy of the document with all sections, see nature.com/documents/nr-reporting-summary-flat.pdf

Ecological, evolutionary & environmental sciences study design

All studies must disclose on these points even when the disclosure is negative.

Study description	This experiment was set up as a fully factorial design containing five levels of the number of global change factors (GCFs) (0, 1, 2, 5, 8 GCFs). There were 20 replicates for the control (zero GCF treatment), 8 for each GCF at single GCF treatment, and 50 replicates for each combined GCF level, for a total of 276 experimental units. The combined GCF treatments were created by randomly selecting GCF from a pool of 12 CGFs.
Research sample	Soils were collected from the top 10 cm of a grassland of Freie Universität Berlin at Albrecht-Thaer-Weg, Berlin (52.28°N, 13.18°E). The co-occurrence of multiple global change factors, including nitrogen deposition, salinity, drought, herbicide, fungicide, antibiotic, insecticide, surfactant, PFAS, heavy metal pollution, microplastic and pesticides, has been reported by recent studies in soil ecosystems. Therefore, we used the field soil of a grassland to test the responses of soil properties and functions to the number and dissimilarity of co-acting multiple global change factors.
Sampling strategy	Sample size was determined based on a previous study with broadly comparable experimental design and materials. In this previous study (Rillig et al 2019, Science, 366, 886-890), we had the following levels of replication: control (n=20), individual factors (n=8 each), and factor richness levels (n=10 each). To obtain larger variation of factor combinations in high factor levels, the number of replicates for each multi-factor level was increased to 50 replicates. The factor combinations in multi-factor levels have been created by a random factor-selection method. That is, firstly, complete sets of factor combinations for each factor level were generated (e.g., for the 5 factor level, there are in total 792 different factor combinations for choosing 5 factors from a 12 factor pool). Then we randomly selected 50 factor combinations from the possible combinations at each factor level without replacement to avoid selecting repeated factor combinations.
Data collection	We measured the following response variables: litter decomposition rate tested by a balance, soil enzyme activity measured using a microplate reader (BioRad, Benchmark Plus, Japan), water-stable soil aggregates tested using a sieving machine (Agrisearch Equipment, Eijkelkamp, Giesbeek, Netherlands) and soil pH tested using a pH meter (Hanna Instrument, Smithfield, USA). Mohan Bi, Huiying Li, Peter Meidl and Yanjie Zhu colleted and recorded data using online spreadhseets.
Timing and spatial scale	This experiment started on 01/03/2022 and stopped on 14/04/2022. In our previous study (Rillig et al 2019), six weeks were sufficient to detect significant responses of soil properties and functions to multiple GCF treatments. Therefore, soils with treatments were incubated for six weeks, and then we sampled all soils and did the measurement in the present study. Spatial scale was not considered in this study, because all 40 g of soil in each experimental unit was used for measurements.
Data exclusions	No data were excluded from the analyses, with the exception of enzyme analysis, we excluded invalid enzyme data (<0).
Reproducibility	There were a large number of replicates, e.g., 50 repeats for the multiple GCF treatments, and 5 gradients of GCF treatments, which lead to a total of 276 experimental units. Furthermore, all experimental units were incubated in a climate chamber with a precise control of temperature. These will ensure the confidence that the detected response trend can be reproduced
Randomization	All treatments were randomly allocated to each experimental unit (Mini Bioreactor). Besides, all Mini Bioreactors were randomly located in the incubation room.

Blinding	Each sample was labeled by a number during sampling and measurement. The number did not indicate a treatment of a sample.

Did the study involve field work? ☒ Yes ☐ No

Field work, collection and transport

Field conditions	We collected field soil from the top 10 cm of a grassland in Albrecht-Thaer-Weg, Berlin (52° 28′ N, 13°18′ E). The soil is an Albic Luvisol and has the following properties: 73.6% sand, 18.8% silt and 7.6% clay; pH 7.1 ($CaCl_2$), 6.9 mg P/100 g soil (calciumacetatelactate), 0.12% nitrogen and 1.87% carbon. The weather conditions are available form a nearby meteorological station (https:// www.agrar.hu-berlin.de/de/institut/departments/dntw/agrarmet/service/wo/Klima-Dahlem.html). Annual mean air temperature for 1991-2020 is 10.4 °C. Mean soil temperature in the growing season from May to October in the 0-10 cm is 20 °C, which was used as the incubation temperature in this experiment. Mean annual precipitation for 1991-2020 is 561.6 mm.
Location	Albrecht-Thaer-Weg, Berlin, Germany (52° 28′ N, 13°18′ E)
Access & import/export	A small amount of soils (24 kg) were collected from the experimental field site of the Freie Universität Berlin, and for this no permit is required.
Disturbance	The collection of 24 kg of soils on a grassland had minor effects on the ecosystem on this dedicated experimental site.

Reporting for specific materials, systems and methods

We require information from authors about some types of materials, experimental systems and methods used in many studies. Here, indicate whether each material, system or method listed is relevant to your study. If you are not sure if a list item applies to your research, read the appropriate section before selecting a response.

Materials & experimental systems

n/a	Involved in the study	
☒	☐	Antibodies
☒	☐	Eukaryotic cell lines
☒	☐	Palaeontology and archaeology
☒	☐	Animals and other organisms
☒	☐	Clinical data
☒	☐	Dual use research of concern
☒	☐	Plants

Methods

n/a	Involved in the study	
☒	☐	ChIP-seq
☒	☐	Flow cytometry
☒	☐	MRI-based neuroimaging

Plants

Seed stocks	NA
Novel plant genotypes	NA
Authentication	NA
